# The mechanism of oleic acid inhibiting platelet activation stimulated by collagen

**DOI:** 10.1186/s12964-023-01276-0

**Published:** 2023-10-10

**Authors:** Xianghui Zhou, Xin Zhou, Ruirui Zhu, Zhangyin Ming, Zhipeng Cheng, Yu Hu

**Affiliations:** 1 Department of Hematology, Union Hospital, Tongji Medical College, Huazhong University of Science and Technology, 1277 Jiefang Avenue, Wuhan, 430022 Hubei Province China; 2 Collaborative Innovation Center of Hematology, Huazhong University of Science and Technology, Wuhan, 430022 China; 3 Department of Stomatology, Union Hospital, Tongji Medical College, Huazhong University of Science and Technology, Wuhan, 430022 China; 4 School of Stomatology, Tongji Medical College, Huazhong University of Science and Technology, Wuhan, 430030 China; 5 Department of Cardiology, Union Hospital, Tongji Medical College, Huazhong University of Science and Technology, Wuhan, 430030 China; 6 Department of Pharmacology, School of Basic Medicine, Tongji Medical College, Huazhong University of Science and Technology, Wuhan, 430030 China; 7 Tongji-Rongcheng Center for Biomedicine, Huazhong University of Science and Technology, Wuhan, 430030 China

**Keywords:** Arterial thrombosis, Oleic acid, Platelet aggregation

## Abstract

**Background:**

Abnormal platelet activation is a key factor in the occurrence and development of thrombotic diseases. However, the physiological mechanisms that underlie platelet homeostasis remain unclear. Oleic acid, one of the most abundant lipids in the human diet, has potential antithrombotic effects. This study aimed to investigate the effects of oleic acid on platelet activation and thrombosis.

**Methods:**

Platelet aggregation, ATP release, and fibrinogen spread were evaluated to determine the role of oleic acid in platelet activation. A ferric chloride-induced carotid injury model was used to establish the effect of oleic acid on thrombus formation in vivo. Western blotting analysis and transfection experiments were performed to determine the mechanisms involved in this process.

**Results:**

Oleic acid inhibited platelet aggregation, granule release, and calcium mobilization. Furthermore, it inhibited the spread of platelets on fibrinogen. We also found that oleic acid delayed arterial thrombosis in mice, as demonstrated in a murine model of ferric chloride-induced carotid artery thrombosis. The molecular mechanism of its inhibition of platelet activity may be through the Syk-PLCγ2 and CaMKKβ/AMPKα/VASP pathways. In addition, we demonstrated that the phosphorylation of AMPK at Ser496 was an important mechanism of platelet activation.

**Conclusions:**

Our study showed that oleic acid inhibits platelet activation and reduces thrombogenesis by inhibiting the phosphorylation of multiple signaling molecules, offering new insights into the research and development of antiplatelet drugs.

Video Abstract

**Supplementary Information:**

The online version contains supplementary material available at 10.1186/s12964-023-01276-0.

## Background

Thrombotic diseases have gradually become one of the main causes of death in humans [1, 2]. The occurrence and development of thrombosis include many factors, including vascular endothelial injury and changes in hemodynamics and blood composition. During hemostasis and thrombosis, platelet activation plays an important role in the formation of blood clots that are involved in heart attack, stroke, and peripheral vascular disease [3]. Platelets are derived from megakaryocytes and are present in the mammalian blood. In addition to their important roles in thrombosis and hemostasis, their roles in angiogenesis, inflammation, tumor growth, and metastasis cannot be ignored. Although platelets are small in size compared with other cells, they have a rich variety of membrane protein receptors, including the leucine-rich repeat sequence family, integrins, GPCRs, and lectin receptor family [4, 5]. Antiplatelet drugs, such as epoxide inhibitors (aspirin), P2Y12 antagonists (clopidogrel, prasugrel), and αIIbβ3 antagonists (ABciximab), which target different receptors on the surface of platelets are widely used, but the current antiplatelet drugs have different degrees of side effects [6]. Therefore, further studies on the mechanisms underlying platelet activation are of great clinical significance.

Non-esterified fatty acids (NEFAs) are important energy sources in human tissues and have various physiological functions such as receptor signal transduction, gene expression, and regulation of systemic energy homeostasis [7]. Oleic acid (OA), an important component of NEFAs, is produced via both dietary and endogenous syntheses. OA is one of the most abundant forms of lipid in the human diet, accounting for up to 95% of normal ingestion. It is the main monounsaturated omega-9 fatty acid formed by stearoyl-CoA desaturase 1 [8, 9]. OA is recognized as a versatile nutraceutical with many cellular functions. For example, it exhibits antioxidant properties that may be related to the synthesis and activity of antioxidant enzymes [10]. OA also has antithrombotic and anti-atherosclerosis effects and reduces cardiovascular disease-related mortality [11–13]. The reason may be related to its ability to reduce the expression of cholesterol transport-related proteins, reduce cholesterol absorption, and reduce the oxidation of low-density lipoprotein [14, 15]. In recent years, the important role of OA in platelet activation and thrombosis has attracted attention. Leticia et al. showed that peanut vegetable oil, which is rich in OA, can reduce platelet aggregation [16]. A study by Turini et al. also found that when participants consumed vegetable oils rich in OA, the level of platelet aggregation decreased under the stimulation of collagen [17]. However, because all previous experiments used OA mixtures, it is impossible to understand the influence of OA on stimulant-induced platelet activity. Therefore, whether OA affects platelet activation and thrombosis and its regulatory mechanisms need to be clarified. In this study, we investigated the function of OA in platelet activation and thrombus formation and evaluated the intracellular pathways.

## Methods

### Preparation of human washed platelets

Washed platelets were prepared as previously described [18]. Blood samples were collected from volunteers in silica vacuum containers containing 1:9 3.8% sodium citrate. Whole blood was centrifuged at 160 *g* for 14 min. Platelet-rich plasma was obtained as supernatant. Platelets were centrifuged for 10 min in platelet-rich plasma at 475 *g* and washed with HEPES-buffered Tyrode’s solution containing 1 µM prostaglandin E1 and 1mM EDTA. Finally, cells were resuspended in HEPES-buffered Tyrode’s solution. Platelet concentration was adjusted according to the experiment, and the platelets were incubated at 37 ℃ for 30 min before each experiment.

### Platelet aggregation and ATP release

The platelet aggregation assay was performed as previously described [19]. Platelets were resuspended in HEPES-buffered Tyrode’s solution. The final platelet concentration was adjusted to 300 × 10^9^/L, and the platelets were transferred to an aggregation tube for preincubation with different concentrations of NEFAs and CaCl_2_ (1 mM) at 37 °C for 5 min. Aggregation tube transmittance was measured using a platelet aggregation instrument for quantification. Platelet secretion was monitored by measuring ATP release using the Chrono-lume reagent (Chrono-Log).

### Platelet spreading

Platelet spreading was performed as described previously [20]. The concentration was adjusted to 20 × 10^9^/L, and the platelets were added to a six-well plate containing slides. Slides were coated with fibrinogen (50 g/mL) or bovine serum albumin. Platelets were incubated with OA or vehicle for 60 min. Platelet suspensions were discarded, fixed with 2% paraformaldehyde, permeabilized with 0.1% Triton, stained with fluorescein isothiocyanate-labeled phalloidin, and incubated for 45 min at 37 ℃. Images were captured using a fluorescence microscope.

### Ferric chloride-induced carotid injury model

A carotid artery injury model was established according to a previously described method [19]. Mice were injected with 5 mg/kg OA into the tail vein. Thirty minutes later, mice were anesthetized with 80 mg/kg pentobarbital sodium and fixed on an operating table to separate the carotid arteries. First, a flow probe was placed to measure the baseline blood flow. A 3 mm filter paper was immersed in 10% ferric chloride and then applied along the outer membrane of the carotid artery for 1 min. After removing the filter paper, the blood flow through the carotid artery was monitored until a stable clot was formed. Occlusion time was defined as the first time point when stable flow interruption was achieved or when 30 min had passed. The changes in blood flow were recorded on a screen. After 8 min, the thrombus was harvested along the carotid vessel, fixed with paraformaldehyde, and embedded in paraffin. Cross sections were stained with hematoxylin and eosin.

### Statistical analysis

Data were analyzed using GraphPad Prism 8.4.2 (La Jolla, CA, USA). The mean and standard error were calculated. Differences were evaluated using two-tailed Student’s t-test or one-way ANOVA. P < 0.05 was considered significant.

## Results

### Effect of OA on human platelet aggregation

**Fig. 1:**
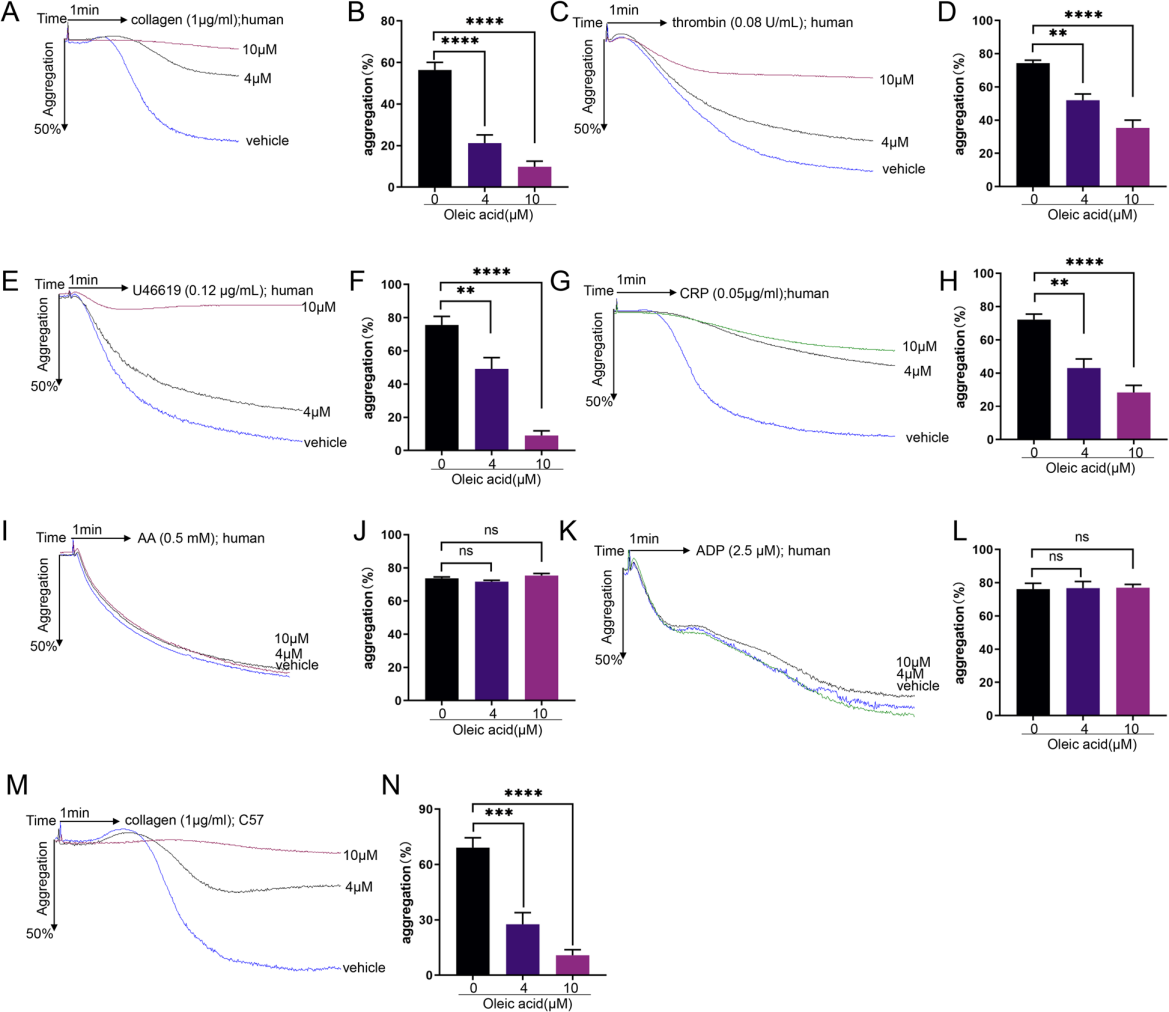
Effect of oleic acid on human platelet aggregation. **A**-**J** Washed platelets from human are pretreated with oleic acid or vehicle for 5 min in the presence of 1 mM CaCl_2_ and stimulated with collagen (1 µg/mL), thrombin (0.08 U/mL), U46619 (0.12 µg/mL), CRP (0.05 µg/mL) and AA (0.5 mM); *N* = 5. **K**-**L** Platelet-rich plasma from human was pretreated with oleic acid or vehicle and stimulated with ADP (2.5 µM); *N* = 5. **M**-**N** Washed platelets from mice were pretreated with oleic acid or vehicle and stimulated with collagen (1 µg/mL); *N* = 5. Data are presented as mean ± standard error of mean; one-way analysis of variance; *p < 0.05, **p < 0.01, ***p < 0.001, ****p < 0.0001, NS indicates no statistical significance

We found that platelet aggregation induced by collagen (1 µg/mL), thrombin (0.08 U/mL), U46619 (0.12 µg/mL), and CRP (0.05 µg/mL) was inhibited by OA (Fig. 1A-H). However, it did not affect arachidonic acid (0.5 mM) and ADP (2.5 µM)-induced aggregation (Fig. 1I-L). Similarly, OA inhibited collagen-stimulated activation of mouse platelets (Fig. 1M-N). Additionally, we examined the role of several NEFAs in collagen-induced platelet aggregation in humans. After incubation with different concentrations of NEFAs for 5 min, the platelets showed an inhibitory effect on platelet aggregation stimulated by collagen. (Supplementary Fig. 1A-E, Additional File 1). The inhibitory effects of different NEFAs differed. We found that palmitic acid had an inhibitory effect. Myristic acid and lauric acid also had an inhibitory effect. However, the inhibitory effect was relatively weak. In addition, stearic acid did not inhibit platelet aggregation during collagen-stimulated platelet activation.

### OA inhibited platelet granule secretion and the αIIbβ3 signaling pathway

**Fig. 2:**
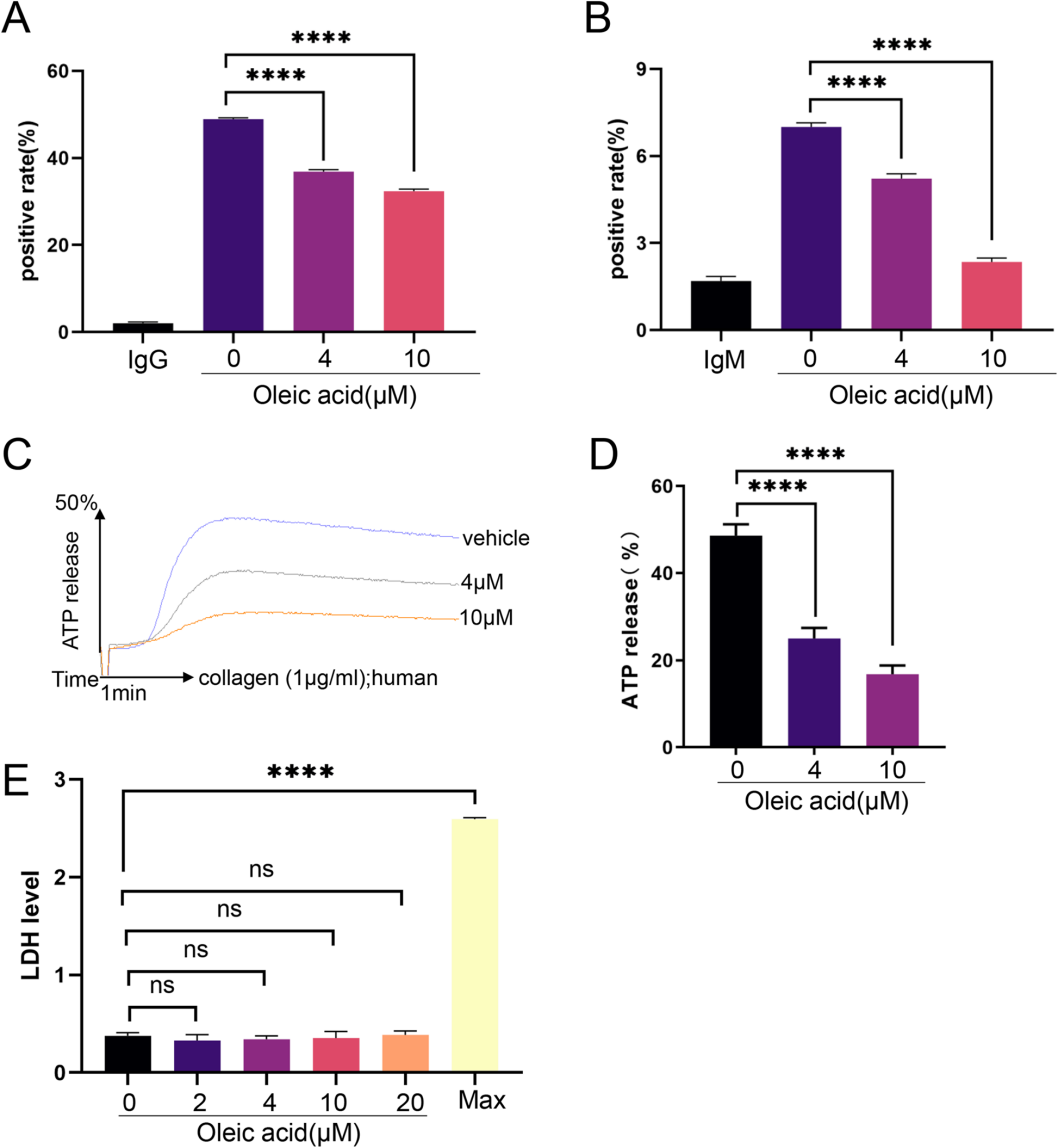
Oleic acid inhibits platelet granule secretion and the αIIbβ3 signaling pathway. **A** Washed human platelets are stimulated with collagen in the presence of various concentrations of oleic acid or vehicle at 37 °C and then labelled with fluorescein isothiocyanate-conjugated P-selectin. P-selectin exposure is determined using flow cytometry; *N* = 5. **B** Washed human platelets are stimulated with collagen in the presence of various concentrations of oleic acid or vehicle at 37 °C and then labelled with fluorescein isothiocyanate-conjugated PAC-1 antibodies. PAC-1 binding is determined using flow cytometry; *N* = 5. **C**-**D** Washed human platelets are pretreated with various concentrations of oleic acid or vehicle at 37 °C and then stimulated with collagen. The concentration of ATP is assessed using Chrono-lume; *N* = 5. **E** Cytotoxicity of oleic acid, as measured using the lactate dehydrogenase assay, is determined in washed platelets treated with oleic acid or vehicle; Max group is the addition of lysate; *N* = 6. Data are presented as mean ± standard error of mean; one-way analysis of variance; *p < 0.05, **p < 0.01, ***p < 0.001, ****p < 0.0001, NS indicates no statistical significance

To detect the effect of OA on platelet granule secretion, platelets were preincubated with OA at 37 ℃ for 5 min, stimulated with collagen (3 µg/mL), and subsequently incubated with the antibody, followed by detection using flow cytometry. These results showed that OA inhibited P-selectin exposure during collagen-stimulated platelet activation (Fig. 2A). In addition, we investigated the effect of OA on the activation of the αIIbβ3 integrin using flow cytometry. As determined by the binding of the PAC-1 antibody, preincubation with OA decreased PAC-1 binding in a dose-dependent manner (Fig. 2B). Thus, OA had a dose-dependent inhibitory effect on collagen-stimulated P-selectin exposure and PAC-1 binding. In addition, through platelet release experiments, we found that OA inhibited the release of ATP stimulated by collagen (1 µg/mL) (Fig. 2C-D). Furthermore, we evaluated the cytotoxic effects of OA on platelets by measuring the LDH release. No significant increase in LDH levels was observed in the platelet supernatant after OA administration (Fig. 2E), suggesting that OA had no cytotoxic effects on platelets at the prescribed dose. Similarly, LDH release levels were measured when platelets were incubated with palmitic, myristic, lauric, and stearic acids (Supplementary Fig. 1F-I, Additional File 1).

### OA inhibited integrin 𝜶IIb𝜷3 outside-in signaling

**Fig. 3:**
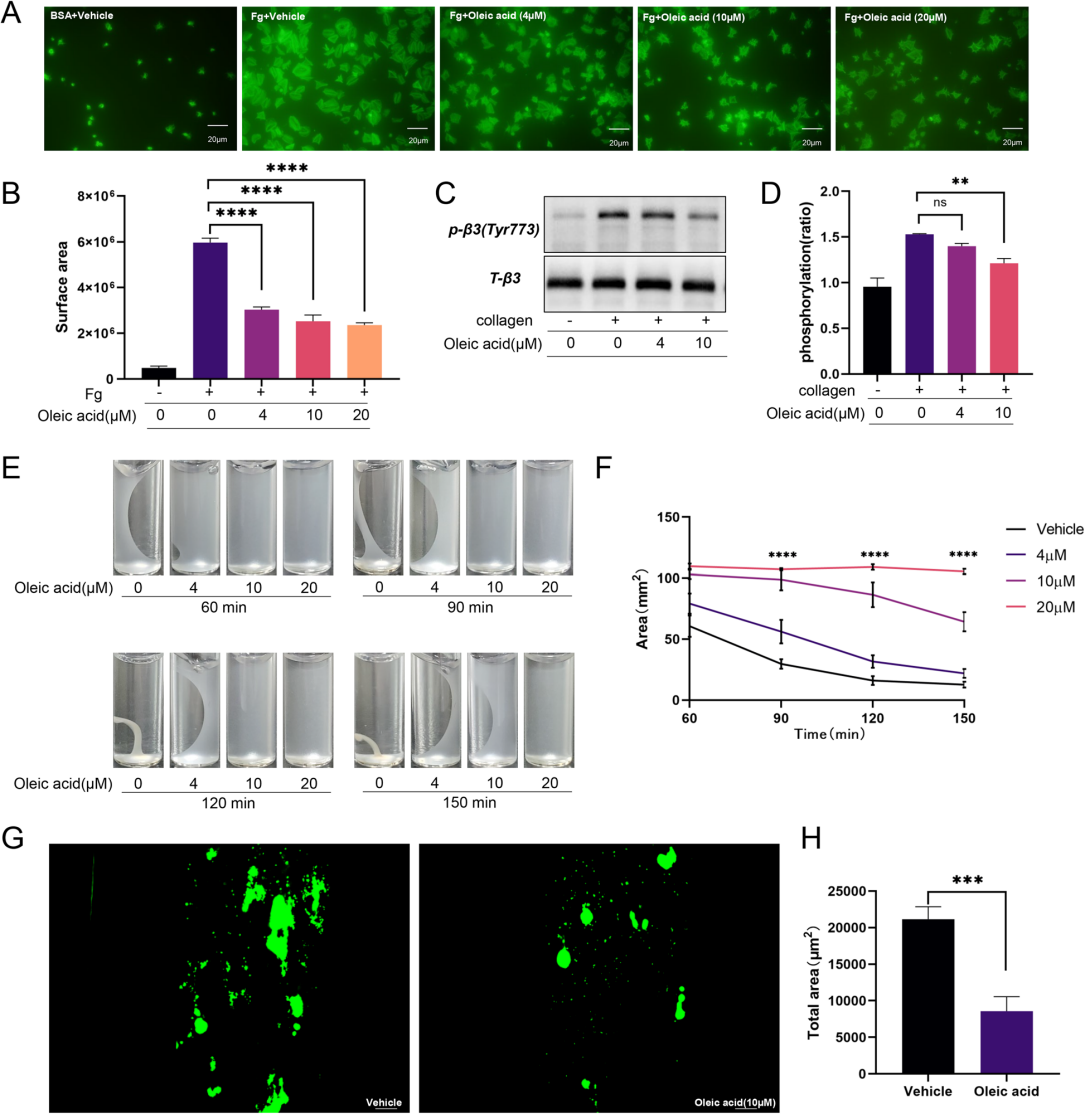
Oleic acid inhibits integrin αIIbβ3 outside-in signaling. **A**-**B** Washed human platelets exposed to immobilized fibrinogen for 60 min are incubated with various concentrations of oleic acid or vehicle. **A** Representative images and (**B**) summary data of the mean platelet surface area are shown; *N* = 5. **C**-**D** Western blotting is performed to assess the phosphorylation levels of integrin 𝛽3 (Tyr773); *N* = 5. **E**-**F** Washed human platelets are incubated with oleic acid or vehicle, and the clot retraction assay is performed; *N* = 5. **G**-**H** Washed human platelets are labelled with Cell Trace Calcein Green and then incubated with vehicle or oleic acid for 5 min. Representative images of surface coverage are shown under arterial shear rates(2000/s). Scale bar = 50 μm; *N* = 8. Data are presented as mean ± standard error of mean; one-way analysis of variance; *p < 0.05, **p < 0.01, ***p < 0.001, ****p < 0.0001, NS indicates no statistical significance

In our study, platelets exposed to bovine serum albumin remained disk-like, whereas platelets exposed to immobilized fibrinogen had a significantly increased surface area. Incubation with OA decreased the surface area of the spread platelets (Fig. 3A-B). To further confirm the effect of OA on outside-in signaling, we measured β3 phosphorylation, an indicator of αIIbβ3 activation. OA was found to inhibit the phosphorylation of β3 (Fig. 3C-D). Platelet retraction is a manifestation of the αIIbβ3 signal transduction. In this study, we found that plaque retraction in platelets treated with OA was inhibited at the same time points (Fig. 3E-F). Moreover, OA reduced the area of platelet adhesion to the collagen matrix under arterial shear conditions (Fig. 3G-H). In addition, platelet adhesion was reduced under venous shear conditions (200/s), and OA also reduced the area of platelet adhesion to the collagen matrix. (Supplementary Fig. 2A-B, Additional File 1).

### OA inhibited intracellular Ca^2+^ concentrations in platelets

**Fig. 4:**
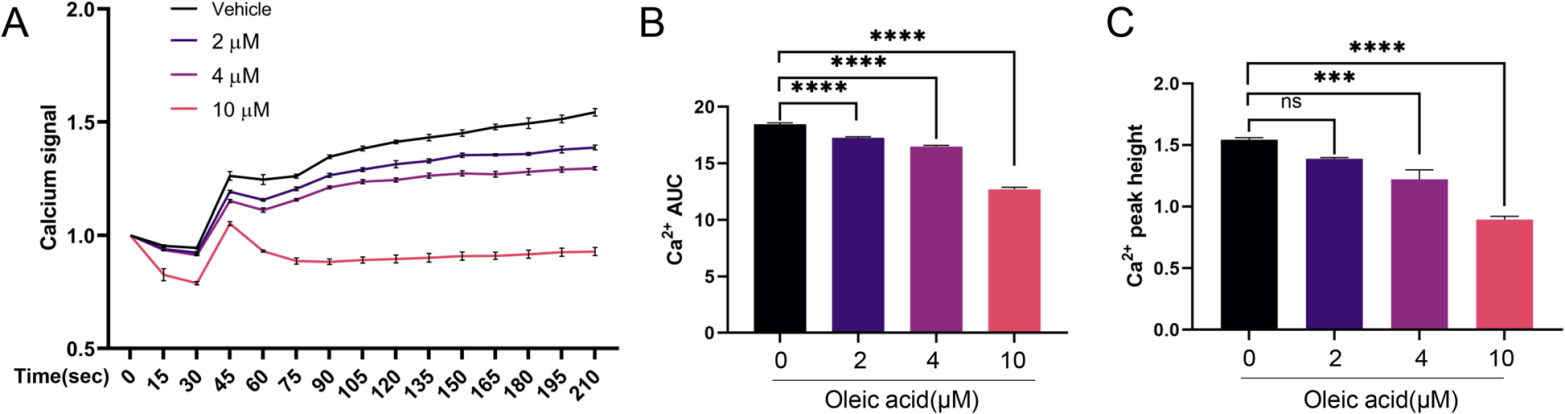
Oleic acid inhibits intracellular Ca^2+^ concentrations in platelets. **A**-**C** Collagen (6 µg/mL) induced Ca^2+^ mobilization, as determined by the Fluo-3-AM fluorescent signal, which is monitored over time using flow cytometry in platelets pretreated with oleic acid or vehicle. **A** Summary data of the fluorescent signal are shown; *N* = 5. **B** Total Ca^2+^ influx (area under the curve) and (**C**) maximum Ca^2+^ signal (peak height) quantified using the kinetics curve; *N* = 5. Data are presented as mean ± standard error of mean; one-way analysis of variance; *p < 0.05, **p < 0.01, ***p < 0.001, ****p < 0.0001, NS indicates no statistical significance

We investigated the effect of OA on collagen-stimulated Ca^2+^ signaling in platelets using Fluo-3-AM. The fluorescence intensity of Ca^2+^ signaling was detected using flow cytometry. As shown in Fig. 4, OA treatment inhibited the Ca^2+^ response. Both the total influx (area under the curve) (Fig. 4B) and the peak Ca^2+^ concentration (peak height) (Fig. 4C) were reduced. Hence, OA suppressed intraplatelet calcium mobilization during platelet activation. In addition, we further examined the effect of OA on collagen-stimulated Ca^2+^ signaling in the presence of EGTA (Supplementary Fig. 2C-D, Additional File 1) or BAPTA-AM (Supplementary Fig. 2E, F, Additional File 1), and found that OA affected calcium mobilization, including intracellular mobilization and extracellular influx.

### OA inhibited thrombosis in vivo

**Fig. 5:**
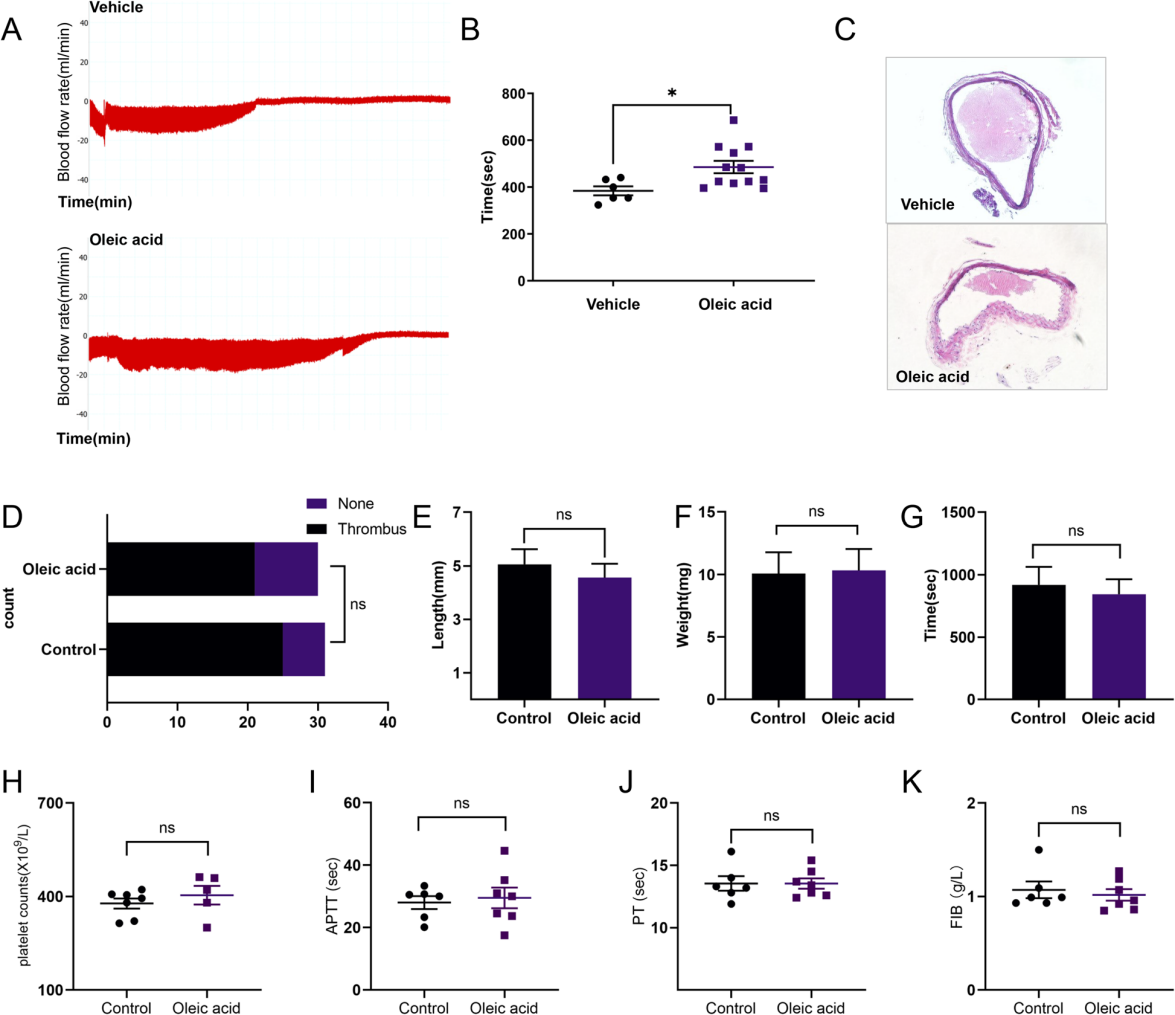
Effect of oleic acid in vivo. **A**-**B** Carotid artery occlusion time is measured after ferric chloride-induced injury using a flow probe. The occlusion time of the oleic acid (5 mg/kg) group is compared with that of the vehicle group. **C** Representative histological images of thrombi stained with hematoxylin and eosin are shown. **D**-**F** (**D**) Venous thrombus formation in mice treated with vehicle or oleic acid (5 mg/kg) (vehicle, 25 out of 31; oleic acid, 21 out of 30); Chi-square test; Furthermore, **E** length and **F** weight of thrombi are compared between the control and experimental groups. **G** The tail bleeding assay is performed in mice treated with oleic acid (5 mg/kg) or vehicle. The tail bleeding time is determined as the time taken for the initial cessation of bleeding; *N* = 8. **H** Mice are injected with oleic acid (5 mg/kg) or the solvent through the tail vein. Thirty minutes later, the whole blood is collected from the orbit, and platelet counts of mice are measured using a blood cell counter (vehicle, *N* = 7; oleic acid, *N* = 5). **I**-**K** Mice are injected with oleic acid (5 mg/kg) or the solvent through the tail vein. The effect of oleic acid on plasma coagulation in mice is detected using activated partial thromboplastin time, prothrombin time, and fibrinogen (vehicle, *N* = 6; oleic acid *N* = 7). Data are presented as mean ± standard error of mean; unpaired t-test; *p < 0.05, **p < 0.01, ***p < 0.001, ****p < 0.0001, NS indicates no statistical significance

In a ferric chloride-induced carotid injury model, mice treated with OA (5 mg/kg) showed prolonged thrombotic occlusion. The occlusion time in the OA group (485.9 ± 90.74 s) was different from that in the control group (384.3 ± 47.55 s) (Fig. 5A-B). H&E staining analysis of carotid thrombosis in mice showed reduced embolism in mice treated with OA (Fig. 5C).

### Effect of OA in venous thrombus and hemostasis

For venous thrombosis, mice were injected with OA or vehicle before and 24 h after surgery. Venous thrombus formation was observed in 21/30 (70%) mice treated with OA and 25/31 (81%) mice treated with vehicle 48 h after IVC stenosis (Fig. 5D). The length and weight of the thrombus were comparable between OA- and vehicle-treated mice (OA: length, 4.563 ± 2.081 mm, *n* = 16; control: length, 5.059 ± 2.351 mm, *n* = 17; *p* = 0.5265 and OA: weight, 10.35 mg ± 6.826 mg, *n* = 16; control: weight, 10.09 mg ± 7.008 mg, *n* = 17; p = 0.9161; Fig. 5E-F). To further determine whether OA could affect physiological hemostasis, tail bleeding experiments were conducted, and it was observed that the tail bleeding time of mice treated with OA did not change significantly (Fig. 5G). In addition, we found that platelet counts in mice injected with OA or the solvent were comparable (Fig. 5H). The blood coagulation index (activated partial thromboplastin time, prothrombin time, and fibrinogen) of mice was determined after injection with OA or vehicle, and no significant effect was observed (Fig. 5I-K).

### OA inhibited Syk-PLCγ2, PI3K-Akt, mitogen-activated protein kinase signal transduction

**Fig. 6:**
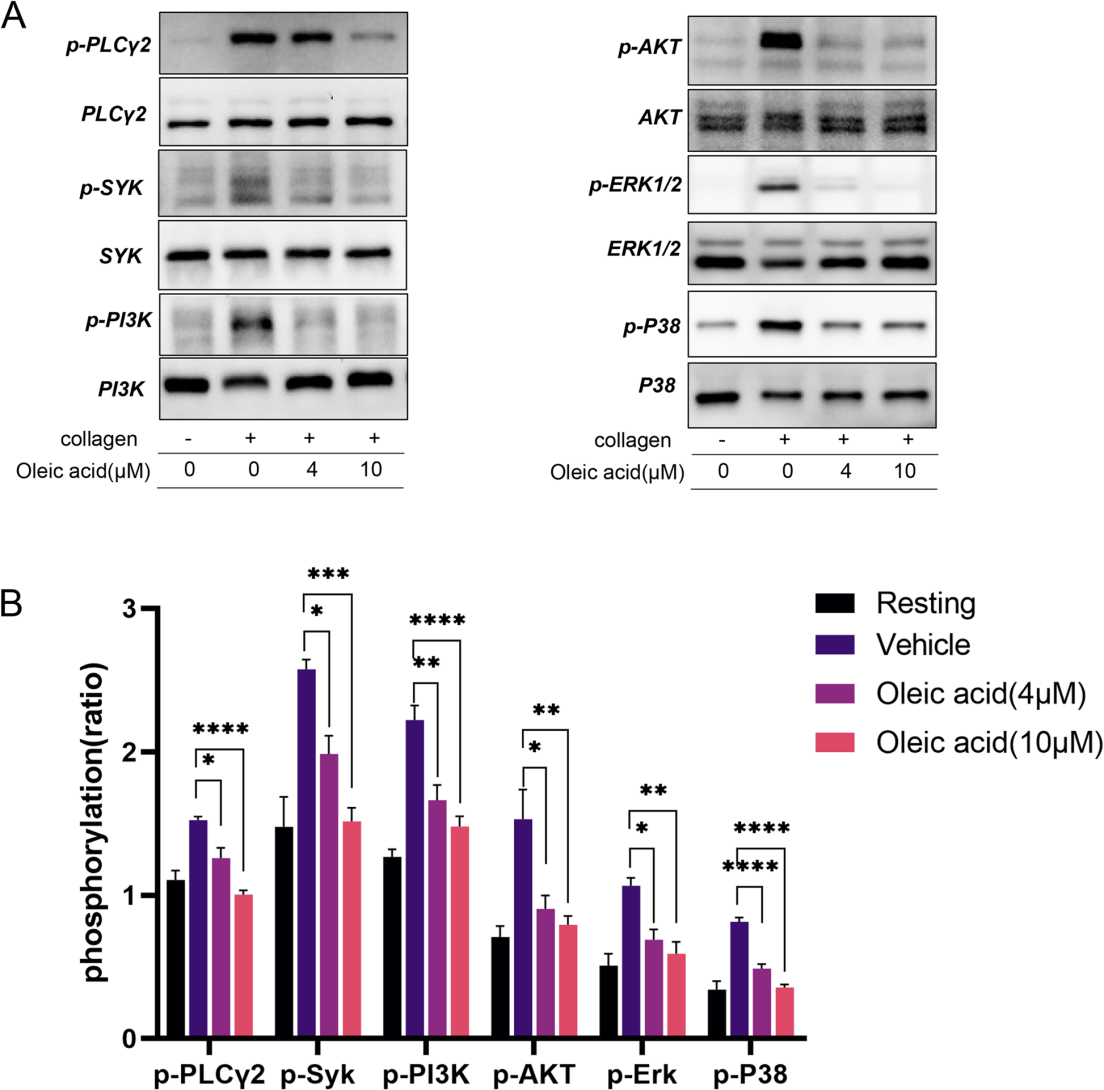
Oleic acid inhibits intracellular platelet signal transduction. **A**-**B** Washed human platelets are pretreated with oleic acid (0 µM, 4 µM, 10 µM), stimulated with collagen, and subsequently lysed with lysis buffer. Western blotting is performed to assess the phosphorylation levels of PLCγ2, Syk, PI3K (Y607), Akt (Ser473), ERK1/2, and p38; *N* = 5. Data are presented as mean ± standard error of mean; one-way analysis of variance; *p < 0.05, **p < 0.01, ***p < 0.001, ****p < 0.0001, NS indicates no statistical significance

To investigate the mechanism of OA inhibiting platelet activation, platelets were incubated with different concentrations of OA for 5 min, and the collagen-stimulated aggregation of the platelet response was terminated at the same reaction time. Denatured protein samples were obtained through a series of treatments. We detected phosphorylation of signaling molecules downstream of the glycoprotein VI (GPVI) signaling pathway, such as Syk-PLCγ2 and PI3K-Akt. We found that OA reduced the phosphorylation of Syk, PLCγ2, Akt, and PI3K (Fig. 6A-B). In addition to Syk/PLCγ2/PI3K/Akt, we found that OA also blocked the phosphorylation of p38 mitogen-activated protein kinase (MAPK) and ERK1/2 in human platelets (Fig. 6A-B). In addition, we also examined the effect of OA on the phosphorylation of intracellular signals in platelets stimulated by thrombin, and found that OA can also inhibit thrombin-stimulated platelet activation by inhibiting the phosphorylation of Syk, PLCγ2, Akt, PI3K, P38, and ERK1/2 (Supplementary Fig. 3, Additional File 1).

### Effect of OA on the calmodulin-dependent kinase kinase β/AMPKα pathway in platelets

**Fig. 7:**
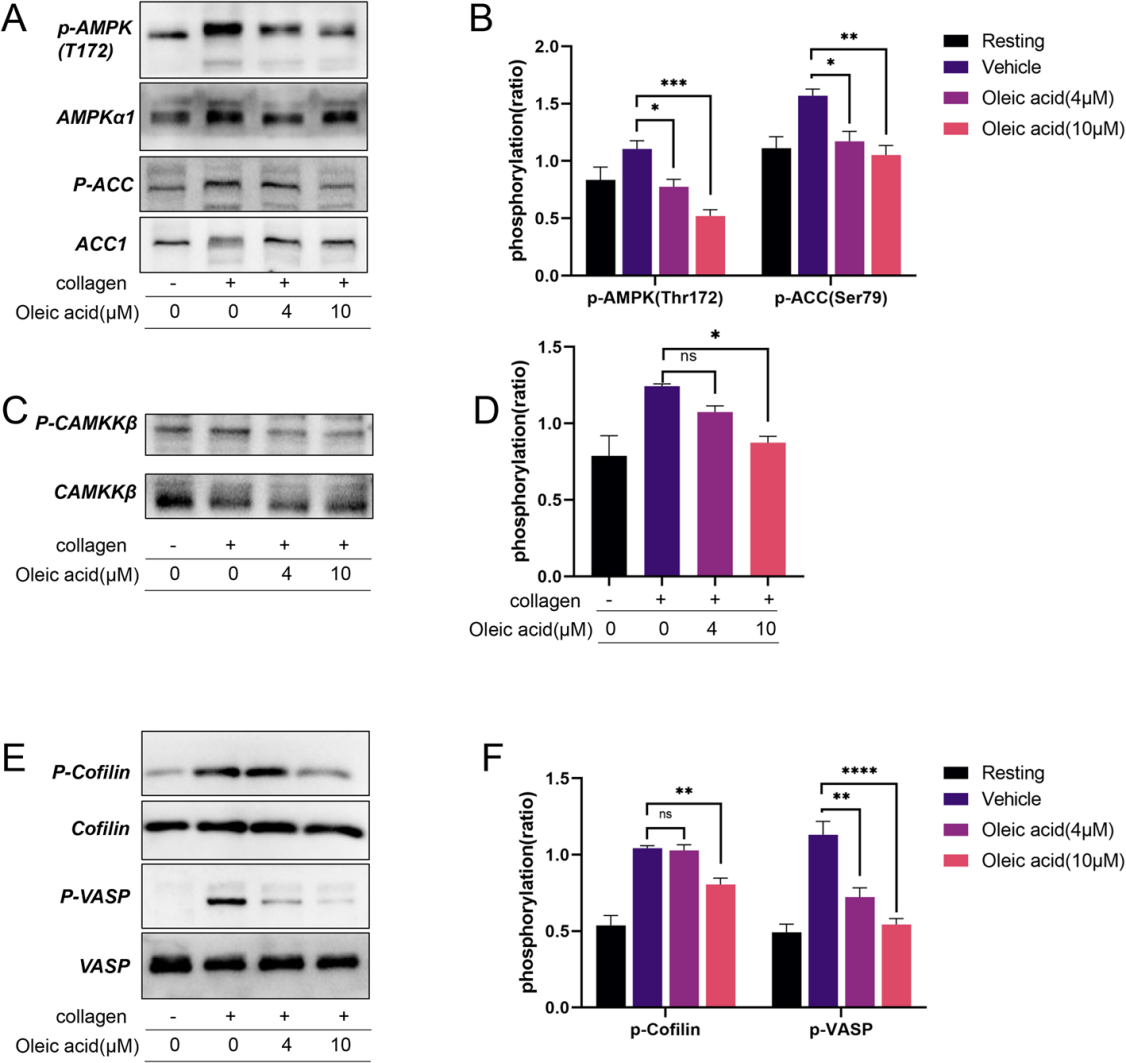
Oleic acid inhibits CaMKKβ/AMPK signal transduction. **A**-**B** Washed human platelets are pretreated with oleic acid (0 µM, 4 µM, 10 µM), stimulated with collagen, and subsequently lysed with lysis buffer, followed by immunoblotting using phospho-AMPK (Thr172) and ACC (Ser79) antibody; *N* = 5. **C**-**F** Western blotting is performed to assess the phosphorylation levels of CaMKKβ, Cofilin (Ser3), and VASP(Thr278), *N* = 5. Data are presented as mean ± standard error of mean; one-way analysis of variance; *p < 0.05, **p < 0.01, ***p < 0.001, ****p < 0.0001, NS indicates no statistical significance

OA inhibited AMPK activation (Fig. 7A). Phosphorylation of AMPKα on Thr172 was inhibited by OA. The effect of OA was dose-dependent. To further evaluate AMPKα activation, we measured the phosphorylation of acetyl-CoA carboxylase (ACC), which is downstream of AMPKα. OA inhibited ACC phosphorylation at Ser79 (Fig. 7A). Because calmodulin-dependent kinase kinase β (CaMKKβ) is an upstream kinase of AMPKα, we speculated that it might be an important pathway protein that mediates OA to inhibit AMPK activation in platelets. Therefore, we determined the phosphorylation level of CaMKKβ and found that OA blocked CaMKKβ phosphorylation (Fig. 7C-D). These data suggest that OA may affect platelet activation through the CaMKKβ/AMPKα pathway under our experimental conditions. The CaMKKβ/AMPKα pathway regulates cytoskeletal organization. Therefore, we evaluated the phosphorylation of cytoskeletal targets downstream of this pathway and found that OA inhibited vasodilator-stimulated phosphoprotein (VASP) and cofilin in platelets. OA decreased cofilin phosphorylation at Ser3 in platelet. In addition, we tested VASP phosphorylation on Thr278, a residue specifically phosphorylated by AMPKα (Fig. 7E-F).

### Relationship between OA and AMPK (Ser496) phosphorylation in platelets

**Fig. 8:**
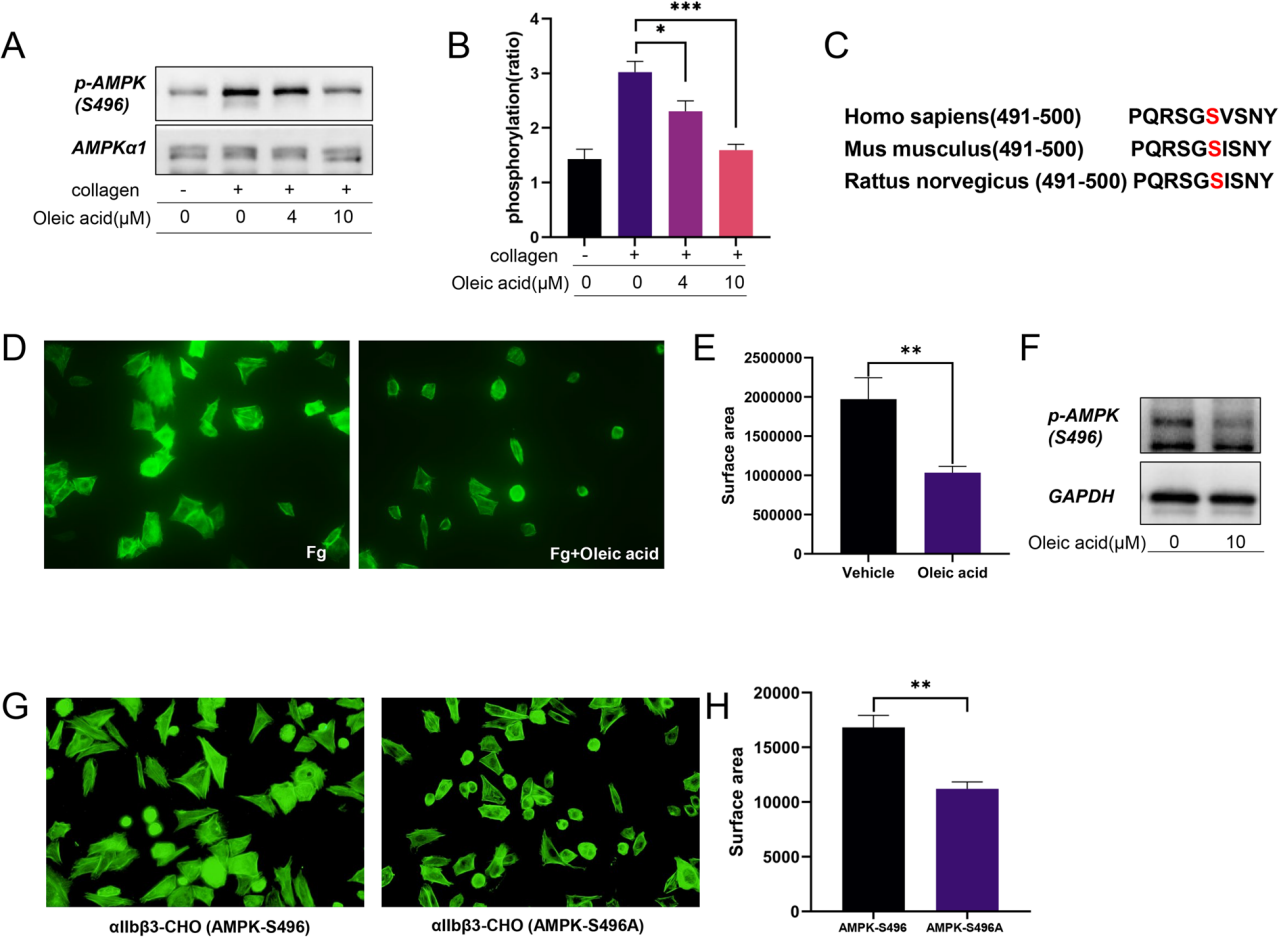
Oleic acid is associated with AMPK (Ser496) phosphorylation in platelets. **A**-**B** Washed human platelets are pretreated with oleic acid (0 µM, 4 µM, 10 µM), stimulated with collagen, and subsequently lysed with lysis buffer, followed by immunoblotting using phospho-AMPK (Ser496) antibody; *N* = 5. **C** Sequence alignment analysis of S496 of AMPKα1 in *Homo sapiens*, *Mus musculus*, and *Rattus norvegicus* **D**-**E** Immunofluorescence images of actin in αIIbβ3-CHO cells spreading on immobilized fibrinogen in the absence or presence of oleic acid; *N* = 5. **F** αIIbβ3-CHO cells are lysed with lysis buffer, followed by immunoblotting with phospho-AMPK (Ser496). **G**-**H** Immunofluorescence staining of actin in αIIbβ3-CHO cells transfected with AMPKα1-S496-flag or AMPKα1-S496A-flag plasmids spreading on immobilized fibrinogen; *N* = 6. Data are presented as mean ± standard error of mean; one-way analysis of variance; *p < 0.05, **p < 0.01, ***p < 0.001, ****p < 0.0001, NS indicates no statistical significance

Phosphorylation of AMPK (Thr172) and AMPK (Ser496) was inhibited by OA (Fig. 8A-B). According to sequence alignment analysis of AMPK (Ser496) in *Homo sapiens*, *Mus musculus*, and *Rattus norvegicus*, we found that it is a highly conserved site (Fig. 8C). In addition, we verified that OA inhibited the spread of αIIbβ3-CHO cells (Fig. 8D-E) and that the phosphorylation of AMPK (Ser496) was inhibited in αIIbβ3-CHO cells (Fig. 8F). Moreover, the spreading area of αIIbβ3-CHO cells transfected with AMPKα1-S496-flag plasmids on fibrinogen was larger than that of cells transfected with AMPKα1-S496A-flag plasmids (Fig. 8G-H).

## Discussion

This study showed that OA has antiplatelet and antithrombotic activities. Moreover, we found that OA regulates aggregation induced by different stimulants differently, which may be due to differences in the signaling pathways of different stimulants. For example, collagen mainly activates intracellular platelet signaling via GPVI, whereas the agonist ADP activates platelets via P2Y1 and P2Y12. Thrombin activation is mediated by membrane receptors PAR-1 and PAR-4. U46619 is a TXA2 receptor agonist, and arachidonic acid acts as a substrate for the synthesis of TXA2 [4, 21, 22]. Platelet-collagen interactions are thought to have the greatest significance in arteries and diseased vessels. In the present study, OA inhibited collagen-induced platelet aggregation. GPVI is the major platelet-activating receptor for collagen [23]. GPVI is required for platelet activation at all shear rates. It is a major collagen receptor that causes signal transduction within platelets and can further lead to the activation of integrins α2β1 and αIIbβ3 and thrombosis [24]. Previous studies have shown that GPVI triggers platelet activation through the immune receptor tyrosine activation motif signaling pathway. This activation regulates various platelet functions including adhesion and aggregation [25]. After combining with ligands (collagen), GPVI gradually aggregates on the surface of the platelets. Src kinase-dependent tyrosine phosphorylation of the immune receptor tyrosine activation motif initiates a signaling pathway involving Syk tyrosine kinases and various adapter proteins, resulting in the activation of PLCγ2 [23, 26]. Activation of PLC leads to the formation of IP3 and DAG, and PLCγ2 is mainly involved in the signal transduction of the platelet GPVI pathway [27, 28]. Our results showed that OA reduced the collagen-activated PLCγ2 phosphorylation, suggesting that OA can inhibit the GPVI-Syk-PLCγ2 signaling pathway. Akt activation may be an important target for antithrombotic therapy because deficient platelet activation has been observed in Akt-knockout mice [29]. In addition, the reciprocal activation of PI3K/Akt and MAPKs has been observed [30]. MAPKs, including ERK1/2, p38MAPK, and JNK1/2, are also involved in collagen-induced platelet activation [31, 32]. These kinases are activated by specific MAPKs, and there is evidence that their activation may be related to the activation of integrin αIIbβ3. Among them, the activation of ERK2 may play an important role in collagen-stimulated platelet secretion and aggregation [32, 33]. Our experimental results indicate that when platelets are activated by collagen, OA-mediated inhibition may be related to ERK1/2 and p38MAPK signaling molecules, which also explains the inhibition mechanism. Thrombin is the most potent activator of platelets. Platelet responses to thrombin are mediated by members of the protease-activated receptor (PAR) family of GPCRs (PAR1, PAR3, and PAR4) [34]. The signaling pathways downstream of PAR in human platelets have been extensively studied, including phospholipase C-β (PLC-β), calcium mobilization, and protein kinase C (PKC) [35]. In addition, ERK1/2, p38 can be also activated by thrombin [32]. In this study, we demonstrated that OA can inhibit the Syk-PLCγ2, PI3K/Akt, and MAPK signaling pathway stimulated by thrombin, suggesting that the downstream signaling pathway of OA inhibiting thrombin stimulation is partially consistent with the GPVI-related signaling pathway. AMPK (a serine/threonine protein kinase) is a vital detector of intracellular energy metabolism. It regulates cellular triglyceride and cholesterol production. AMPK phosphorylation abates free fatty acid-mediated de novo lipogenesis and hepatic lipid build-up. A previous study showed that OA decreases AMPK and ACC phosphorylation to a certain extent in primary hepatocytes [36]. In addition, the hypothesis that AMPK is involved in clot retraction and thrombus stability was proposed in a previous study [37]. However, little is known about the interaction between OA and AMPKα1 in the regulation of platelet function. In our study, we found that the activity of AMPK in platelets increased after collagen stimulation, as confirmed by the increased phosphorylation of a threonine residue (Thr172) within the activation loop of its kinase domain. Moreover, preincubation with OA reduced the phosphorylation of AMPKα1 (Thr172), suggesting that the inhibition of platelet activation by OA may occur through the AMPK signaling pathway. ACC is an AMPK substrate, whose phosphorylation state is used to evaluate AMPK activation. Studies have shown that AMPK inhibits ACC1 activity by phosphorylating ACC1 at Ser79, [38] which was confirmed in our study.

Intracellular Ca^2+^ signaling is a major feature of platelet activation and thrombosis. Platelet Ca^2+^ signaling and activation could be both induced by thrombin and collagen which acts via G-protein coupled receptors [39]. In this study, we used calcium chelating agents EGTA and BAPTA-AM, demonstrated that OA could inhibit not only intracellular Ca^2+^ mobilization, but also extracellular influx during platelet activation, which is mutually confirmed with AMPK activation by increasing Ca^2+^ concentrations [40]. Furthermore, one of the major upstream kinases of AMPK has been identified as Ca^2+^/CaMKKβ, which can phosphorylate AMPK at Thr172, and CaMKKβ can be activated by increases in intracellular Ca^2+^ levels [40]. Previous studies on endothelial cells have shown that the activity of CaMKKβ is enhanced by thrombin, thereby promoting AMPK activity [41]. Therefore, we speculated that CaMKKβ was also an important factor affecting AMPK activity during collagen stimulation in platelets. In our study, we confirmed that the phosphorylation level of CaMKKβ in platelets was increased by collagen stimulation, whereas its level decreased after incubation with OA. However, Onselaer et al. [42] found that the use of the CaMKKβ inhibitor STO-609 did not affect collagen-induced platelet aggregation, which seems to contradict our research results. The inconsistent results may be due to the incubation time of the complex molecules and the dosage of agonists used.

Cofilin is a key protein that regulates actin remodeling and is an essential, ubiquitously expressed, and highly conserved actin-binding protein. Its phosphorylation at Ser3 inhibits the binding of actin filaments and filament severing and depolymerization [43]. During secretion/aggregation, cofilin is characterized by cyclic dephosphorylation/phosphorylation, which promotes actin remodeling and generation of freebarbed ends for lamellipodium assembly during platelet activation. VASP regulates actin polymerization. Its anti-capping activity promotes filopodial formation and critically contributes to the platelet aggregation response [44]. Previous studies have shown that the CaMKKβ-AMPKα1 pathway could affect the activity of cofilin, controlling actin cytoskeletal contraction and organization in platelets. AMPK is also recognized as a VASP kinase responsible for Thr278 phosphorylation [42]. In our study, we found that platelet phosphorylation levels of cofilin and VASP increased under collagen stimulation, whereas OA inhibited their phosphorylation. Moreover, AMPK-mediated VASP phosphorylation is necessary for the proper alignment of contractile ventral stress fibers, thereby halting stress fiber elongation and ensuring proper contractility [45]. In consequence, we found that this altered cytoskeletal response was associated with slower and less effective clot retraction in platelets treated with OA than in platelets of the control group. These results indicate that OA may affect the activity levels of cofilin and VASP through the CaMKKβ-AMPKα1 signaling pathway in the downstream GPVI pathway, thus controlling platelet activation.

In addition to the phosphorylation of Thr172, the phosphorylation of the ST-loop has emerged as an important site for the regulation of AMPK by other kinases [46]. Examples of this include phosphorylation of Ser496 in AMPKα1. Liu et al. reported that the phosphorylation of Ser496 in AMPKα1 facilitates mitochondrial fatty acid β-oxidation [47]. Moreover, a study reported that lipid oxidation contributes to at least one-third of the total oxygen consumed by the mitochondria. During platelet activation, the energy requirement spikes and lipid oxidation increases [48]. This could explain our finding that the phosphorylation of AMPK (Ser496) increased when collagen stimulated platelet activation. OA inhibited AMPK (Ser496) phosphorylation, suggesting that this mechanism may be related to reduced mitochondrial metabolism.

Platelets express many integrins, among which the most abundant integrin αIIbβ3 plays an important role in platelet adhesion, aggregation, and thrombosis. In general, αIIbβ3 is usually in a resting state, but after activation, there is a conformational change and it is transformed into a high-affinity activated state, generating a unique and specific ligand-binding site for fibrinogen, von Willebrand factor, and many matrix proteins containing RGD-like sequences [49]. When the conformation of integrin αIIbβ3 changes, PAC-1 can recognize this special conformation and reflect the activation state of integrin. In our study, we found that after co-incubation with OA, the binding of PAC-1 was reduced, that is, the activation of integration was reduced [50]. In addition, ligand binding to αIIbβ3 mediates platelet secretion, stable adhesion, and clot recovery, triggering a series of intracellular signal transduction (“outside-in” signaling). This signal transduction leads to tyrosine phosphorylation of many proteins, such as integrin β3 at the cytoplasmic tail site Tyr773, which is also a marker of outside-in signal transduction in platelets [51]. In our study, we demonstrated that OA inhibits platelet spreading through αIIbβ3 inside-out and outside-in signaling. Vascular endothelial injury is the main cause of platelet adhesion and aggregation in vivo and is an important process in thrombosis. In a mouse model of arterial thrombosis, treatment with OA extended arterial occlusion times. However, we did not find that OA had a significant inhibitory effect on venous thrombosis in the IVC thrombosis model, with no statistical difference in the size and weight of thrombus formation between OA- and solvent-injected mice. These data also support the conclusion that platelet aggregation is a more important factor in arterial thrombosis than in venous thrombosis. Moreover, we found that OA had no significant regulatory effect on bleeding time or coagulation in mice in the tail bleeding and coagulation factor tests, indicating that OA did not affect physiological hemostasis at this dose.

## Conclusion

We demonstrated that OA can inhibit platelet aggregation. The molecular mechanism involved the Syk-PLCγ2 and CaMKKβ/AMPK/VASP signaling pathways. In addition, we demonstrated that the phosphorylation of AMPK at Ser496 is an important mechanism of platelet activation. These results improve our understanding of the association between OA and thromboembolism, and form the basis for the development of antiplatelet drugs.

### Supplementary Information

**Additional file 1.**

## Data Availability

All data generated during this study are included in this article.

## Abbreviations

NEFA: Non-esterified fatty acid
OA: Oleic acid
IVC: Inferior vena cava
LDH: Lactate dehydrogenase
ACC: Acetyl-CoA carboxylase
VASP: Vasodilator-stimulated phosphoprotein
GPVI: Glycoprotein VI
MAPKs: Mitogen-activated protein kinase
CaMKKβ: Calmodulin-dependent kinase kinase β
