# Supplementary material for: The mechanism of oleic acid inhibiting platelet activation stimulated by collagen
Source: Cell Commun Signal. 2023 Oct 10;21:278. doi: 10.1186/s12964-023-01276-0 (PMC10563249; doi:10.1186/s12964-023-01276-0)

**Materials and Methods**

**Study subjects**

**human**

This study recruited healthy volunteers without hematological diseases, cold, and no medication for 2 weeks.

**Animals**

Male C57BL/6 mice were provided by Hubei Experimental Animal Research Center. Mice were reared under standard laboratory conditions controlled at 22-24℃, 12-hour dark/light cycle. All mice were given normal feed and water and acclimated for a week before being used in the experiment.

**Materials**

FITC-labeled phalloidin, EDTA were obtained from Sigma-Aldrich. Collagen was purchased from ChronoPar Aggregation Reagents (Chrono-Log Corporation, Havertown, PA, USA). Fluo 3-AM was obtained from Dojindo Laboratories (Kumamoto 861-2202, Japan). PGE1, Oleic acid (HY-N1446), Lauric acid (HY-Y0366), Palmitic acid (HY-N0830), Stearic acid (HY-B2219), Myristic acid (HY-N2041), were purchased from Med Chem Express (Monmouth Junction, NJ). BAPTA-AM (T6245) was obtained from Topscience. Protease and phosphatase inhibitor cocktails were obtained from Roche (Indianapolis, IN, USA). phoshpo-AMPK (Thr172), phoshpo-Syk(Tyr525/526), phospho-ERK1/2, phospho-Cofilin(Ser3), phospho-CaMKKβ(Ser511), phospho-Akt (Ser473), phospho-p38 MAPK, and phoshpo-PLCγ2 were obtained from Cell Signaling Technology. phospho-VASP (Thr278) was from ECMBioscience and phospho-AMPK (Ser496) was purchase from Huangzhou HuaAn Biotechnology. Fluorescein isothiocyanate (FITC)-conjugated anti-P-selectin (CD62P) antibodies and FITC-conjugated anti-αIIbβ3 antibodies (PAC1) were from BD Biosciences. Anti-PI3Kinase p85 alpha (phosphoY607) antibody was purchased from Abcam. Collagen-related peptide(CRP) was obtained from Professor Fang Chao's group (Department of Pharmacology, School of Basic Medicine, Tongji Medical College of Huazhong University of Science and Technology)

**Methods**

**Flow cytometric** **analysis**

Platelets (100 × 10^9^/L, 250 µL) were incubated with NEFAs for 5 min prior to activation with Collagen for 5 min at 37 °C. Next, platelets were co-incubated with FITC-conjugated anti-CD62P or anti-PAC-1 antibodies for 15 min in the dark, and cells were collected by BD biological science flow cytometry for analysis.

**Clot retraction analysis**

The platelet concentration was adjusted to 300 × 10^9^/L. Clot retraction was measured in glass tubes. Platelet suspension (500 µL) was added to each tube. Fibrinogen and CaCl_2_ (final concentrations were 400 µg/mL and 1 mM, respectively) were added and pre-incubated with different concentrations of OA or vehicle. Finally, thrombin (0.4 U/mL) was added and incubated at 37℃. Platelet retraction was recorded over time and images were obtained.

**Flow chamber experiments**

Washed human platelets were (25 × 10^9^/L) incubated with the fluorescent dye Cell Trace Calcein Green at 37 °C for 30 min and then added to a Bioflux plate (Bioflux 1000Z; Fluxion Biosciences Inc., USA). The Bioflux plate was coated with 100 µg/mL collagen and incubated overnight at 4 °C. The labeled platelets were perfused through Bioflux plates at a wall shear rate of 2,000/s or 200/s for 10 min. The Bioflux Montage software (Fluxion Biosciences Inc.) was used to assess platelet coverage.

**Inferior vena cava thrombosis model**

Male C57BL/6 mice were prepared as described above. 30 min before surgery, the experimental and control groups were treated with OA (5 mg/kg) and vehicle, respectively. Mice were anesthetized with pentobarbital sodium (80 mg/kg) administered via intraperitoneal injection. A laparotomy was performed at the midline of the abdomen to expose the inferior vena cava (IVC) and separate it from the surrounding tissue. The side branches of the IVC were ligated. The IVC was then ligated over a 0.3-mm silver wire using an 8–0 polypropylene suture. The wire was subsequently removed, leading to IVC stenosis and marked blood flow restriction. After IVC stenosis, 0.2 mL of 1% ampicillin was injected into the abdominal cavity of mice to prevent infection. The peritoneum was closed using a 7–0 polypropylene suture, and the skin was closed using a 6–0 polypropylene suture. Twenty-four hours after the operation, OA (5 mg/kg) or vehicle was injected into the tail veins of the experimental and control groups, respectively. Thrombectomy was performed 48 h after the operation to observe venous thrombosis.

**Tail bleeding time**

The mice were treated with oleic acid (5 mg/kg) or vehicle. 30 min later, 2 mm distal segment of the mouse tail was excised. The injured tail was gently touched on filter paper immediately at 30 s intervals until the bleeding completely stopped. The bleeding time were recorded.

**Lactate dehydrogenase assay**

Cytotoxic assay kits were used to measure lactate dehydrogenase (LDH) levels in the platelets. Human platelets (300 × 10^9^/L) were prepared and incubated with OA, lauric acid, palmitic acid, stearic acid, myristic acid, or the solvent at 37 °C for 5 min. The supernatant (120 µL) was collected by centrifugation and transferred to a 96-well plate. The prepared detection buffer (60 µL) was added to each well, and the plate was incubated at room temperature for 30 min in the dark. Absorbance was measured at 490 nm.

**Calcium signaling**

Washed human platelets were labelled with 1 µM Fluo-3AM at 37 °C for 30 min, and then incubated with oleic acid or vehicle for 5 min with/without EGTA. After 45s of baseline reading, Platelet agonist collagen (6 µg/mL) and calcium chloride were added into the cell sorting tube, and the fluorescence intensity was recorded by BD flow cytometry.

**Plasmid constructs and transfection**

Plasmids were constructed using standard genetic procedures. AMPKα1 cDNA (PRKAA1(NM_006251)) was cloned into a CMV-MCS-IRES-mCherry-SV40-Neomycin vector. AMPKα1-S496 was mutated to alanine based on p-CMV-flag-AMPKα1. According to the manufacturer’s instructions, αIIbβ3-CHO cells were transfected with the constructed plasmid, P3000 reagent, and Lipofectamine3000 (Invitrogen, CA, USA). αIIbβ3-CHO cells provided a model system for studying αIIbβ3-mediated platelet activation. Cells expressing flag-tagged AMPKα1 and flag-tagged AMPKα1-S496A were obtained 48 h after transfection.

**Western blotting analysis**

Platelet activation was terminated by adding RIPA lysates containing protease and phosphatase inhibitors to the aggregation tube. Then, samples were boiled at 100 °C for 10 min with loading buffer. The obtained protein was separated by 10% SDS-PAGE, then transferred to PVDF membrane, sealed with 5% BSA for 2 h. Next, the membranes were washed with TBST for 3 times, and incubated with primary antibody on a 4℃ shaker overnight. On the second day, the PVDF membrane placed at 4 degrees was taken out, washed with TBST, then incubated with the secondary antibody at room temperature for 1h. Finally, the membranes were washed with TBST for 3 times. Immunoreactive bands were visualized using the Bio-Rad Gel imaging system.


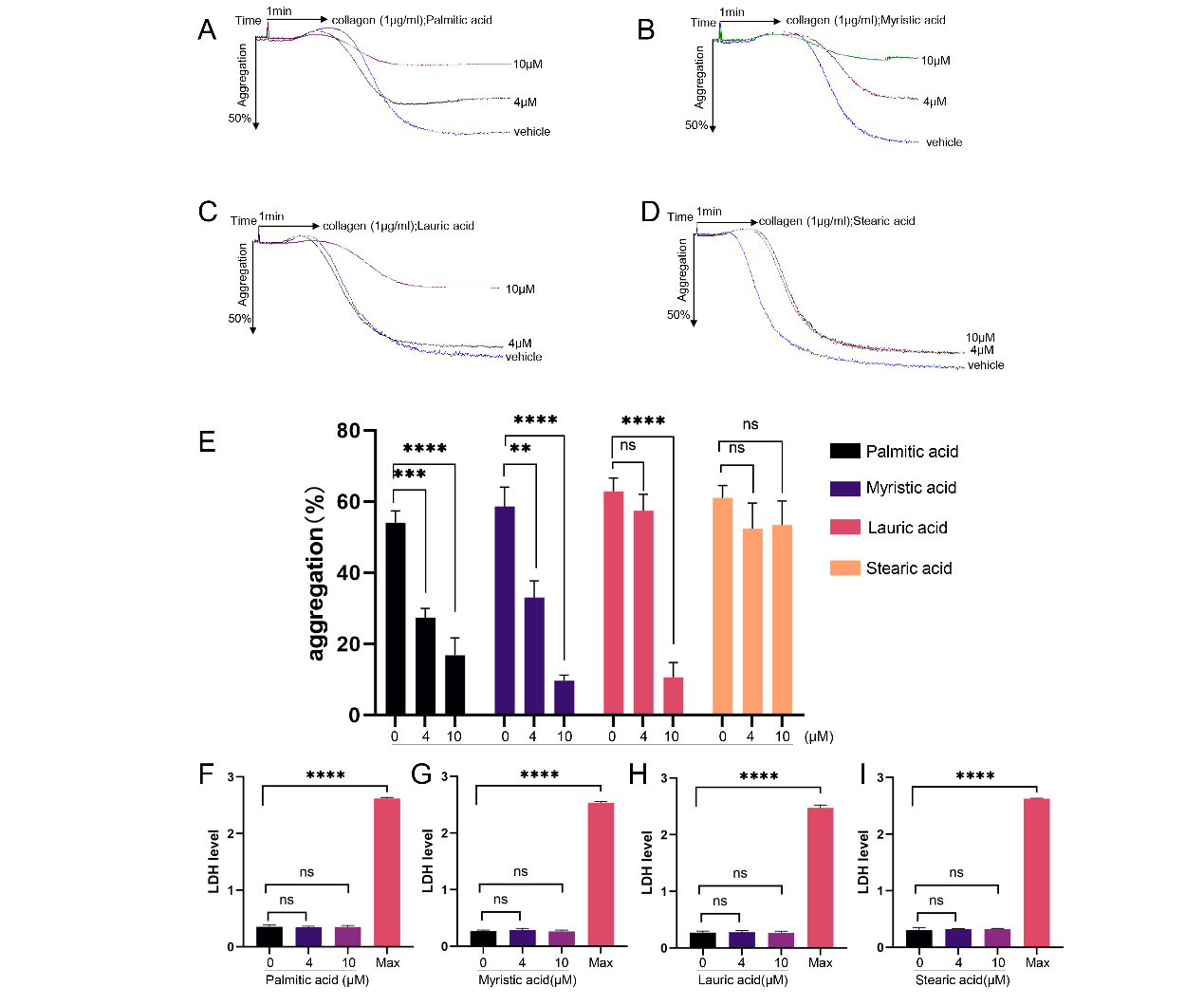


**SUP-Figure 1:** Effect of NEFAs on human platelet aggregation.

(A-D) Washed platelets from humans are pretreated with NEFAs (Palmitic acid; Myristic acid; Lauric acid; Stearic acid) or vehicle for 5 min in the presence of 1 mM CaCl_2_ and stimulated with collagen, and (E) the statistical graph of aggregation; N = 5. (F-I) Cytotoxicity of NEFAs as measured by LDH is determined in washed platelets treated with NEFAs (Palmitic acid; Myristic acid; Lauric acid; Stearic acid) or vehicle, Max group is the addition of lysate; N=6. Data are presented as mean ± standard error of mean; one-way analysis of variance; *p < 0.05, **p < 0.01, ***p < 0.001, ****p < 0.0001, NS indicates no statistical significance.


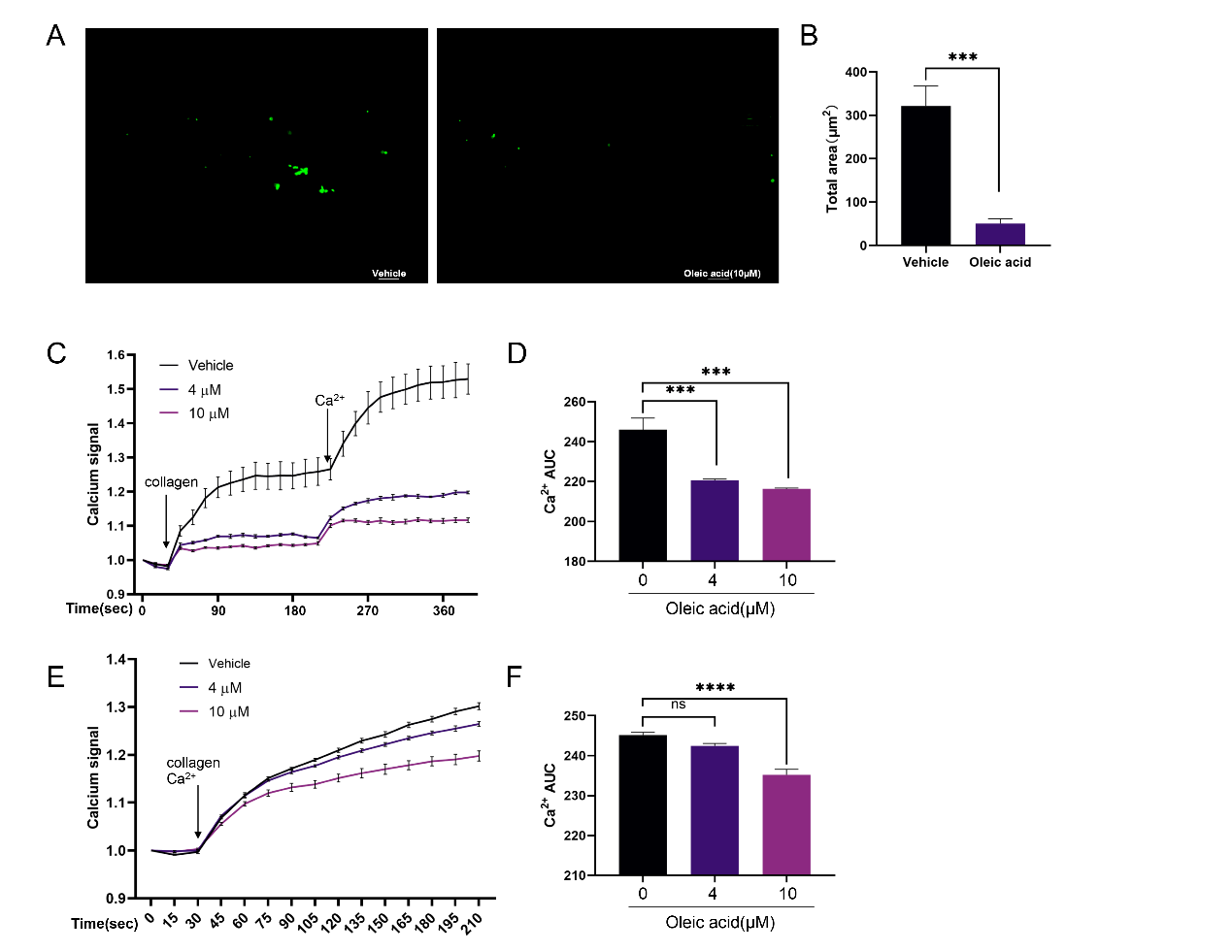


**SUP-Figure 2:** Effect of OA on platelet adhesion and calcium mobilization.

(A, B) Washed human platelets are labelled with Cell Trace Calcein Green and then incubated with vehicle or oleic acid for 5 min. Representative images of surface coverage are shown under venous shear rates (200/s). Scale bar = 50 µm; N = 6. (C, D) Washed human platelets are labelled with Fluo-3-AM and then incubated with vehicle or oleic acid for 5 min in the presence of EGTA. Flow cytometry was used to detect calcium mobilization when collagen stimulated platelet activation; Total Ca^2+^ inflow (area under the curve) was recorded for the first 210 seconds; N=5. (E, F) Washed human platelets are labelled with Fluo-3-AM and then incubated with vehicle or oleic acid for 5 min in the presence of BAPTA-AM. Flow cytometry was used to detect calcium mobilization when collagen stimulated platelet activation; N=5. Data are presented as mean ± standard error of mean; one-way analysis of variance; *p < 0.05, **p < 0.01, ***p < 0.001, ****p < 0.0001, NS indicates no statistical significance.


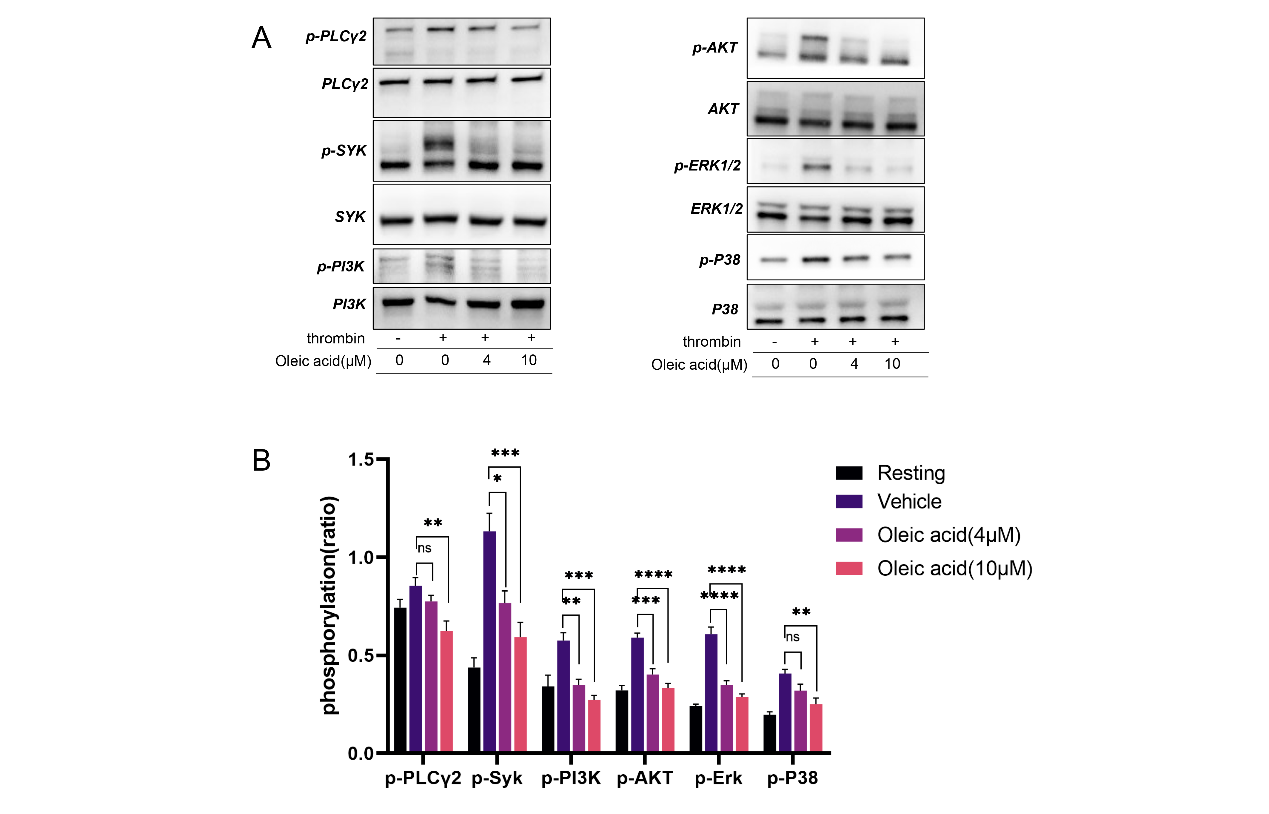


**SUP-Figure 3:** Oleic acid inhibits intracellular platelet signal transduction.

(A, B) Washed human platelets are pretreated with oleic acid (0 µM, 4 µM, 10 µM), stimulated with thrombin, and subsequently lysed with lysis buffer, followed by immunoblotting using phospho-PLCγ2 (Tyr759), Syk (Tyr525/526), PI3K (Y607), Akt (Ser473), ERK1/2, and p38 antibodies; N = 5. Data are presented as mean ± standard error of mean; one-way analysis of variance; *p < 0.05, **p < 0.01, ***p < 0.001, ****p < 0.0001, NS indicates no statistical significance.

**Origin western blots**

**Full unedited gel for Figure 3C**


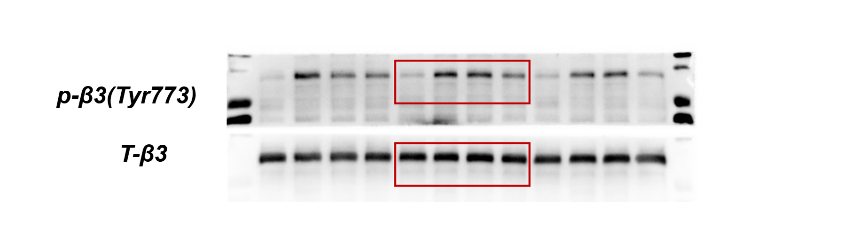


**Full unedited gel for Figure 6A**


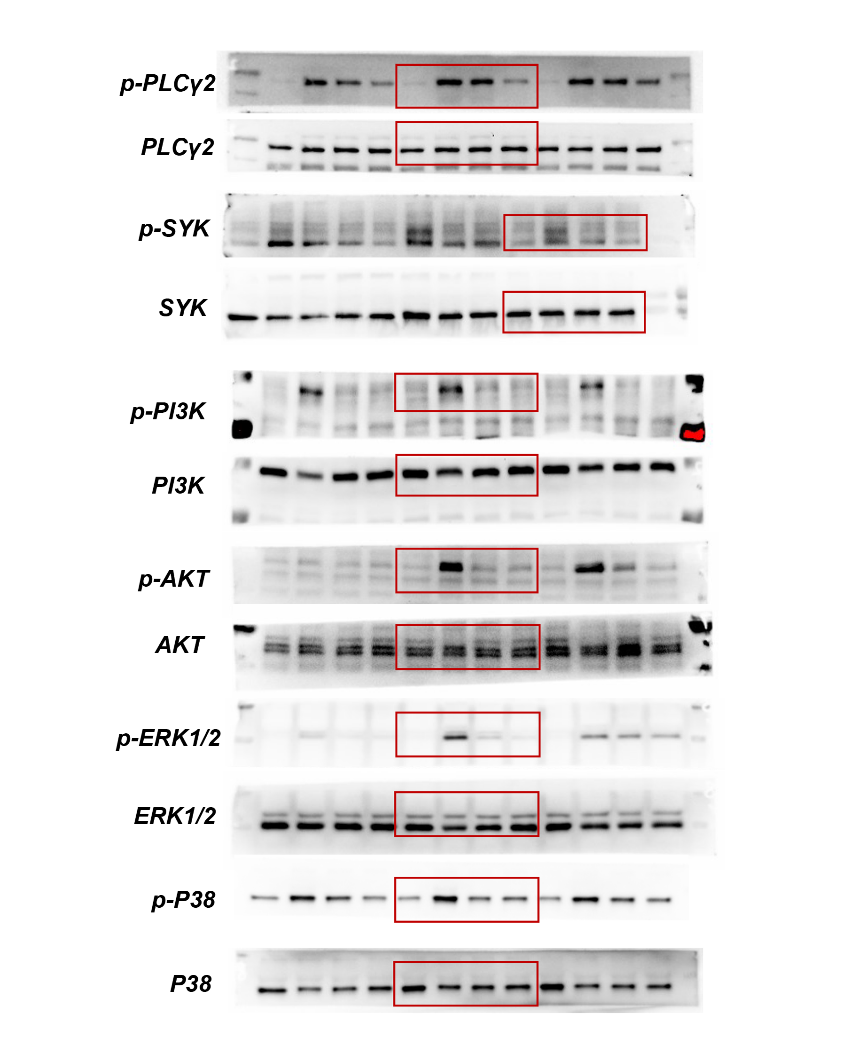


**Full unedited gel for Figure 7A**


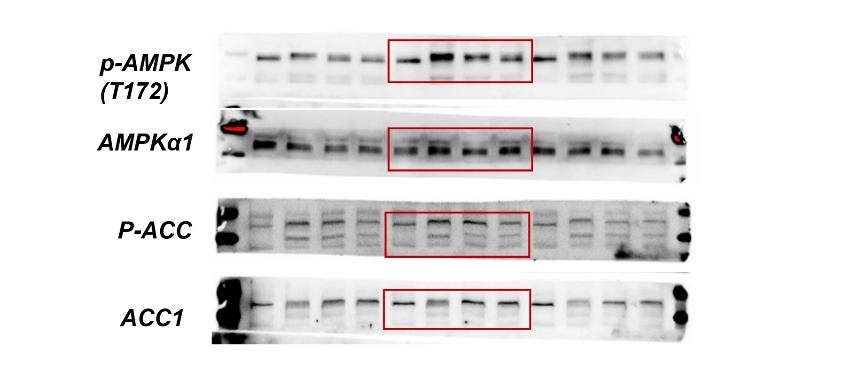


**Full unedited gel for Figure 7C**


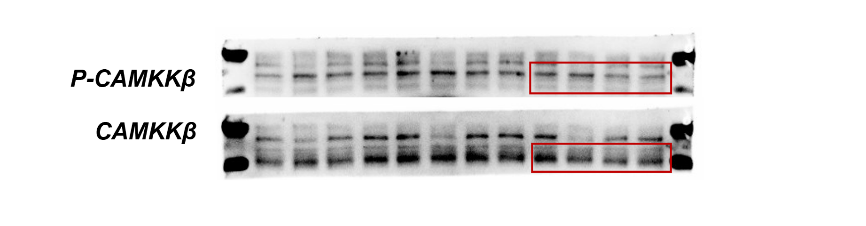


**Full unedited gel for Figure 7E**


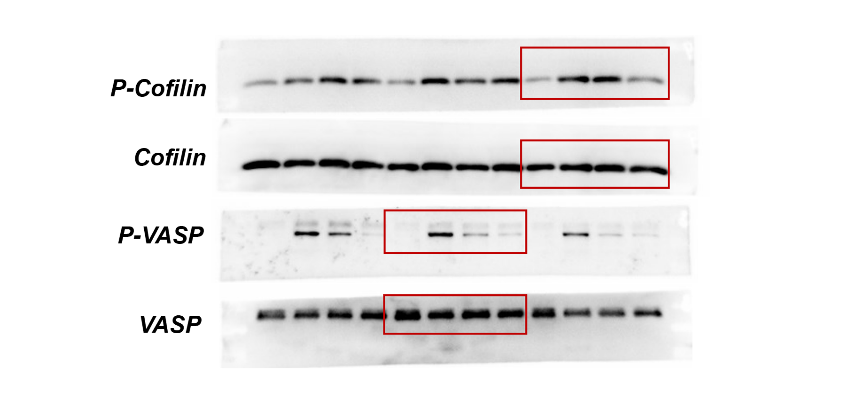


**Full unedited gel for Figure 8A**


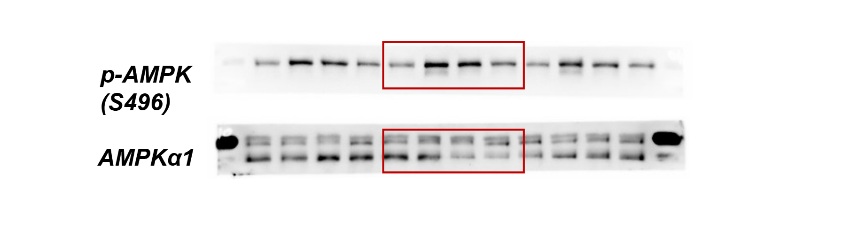


**Full unedited gel for Figure 8F**


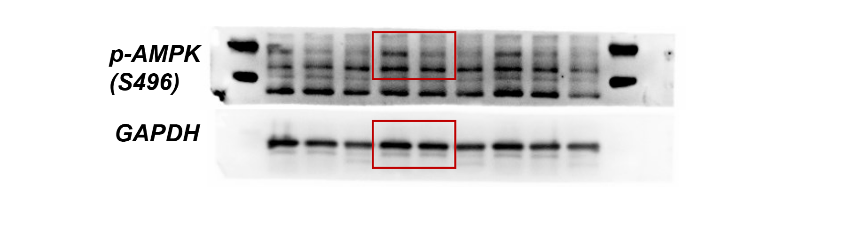


**Full unedited gel for SUP-Figure 3A**


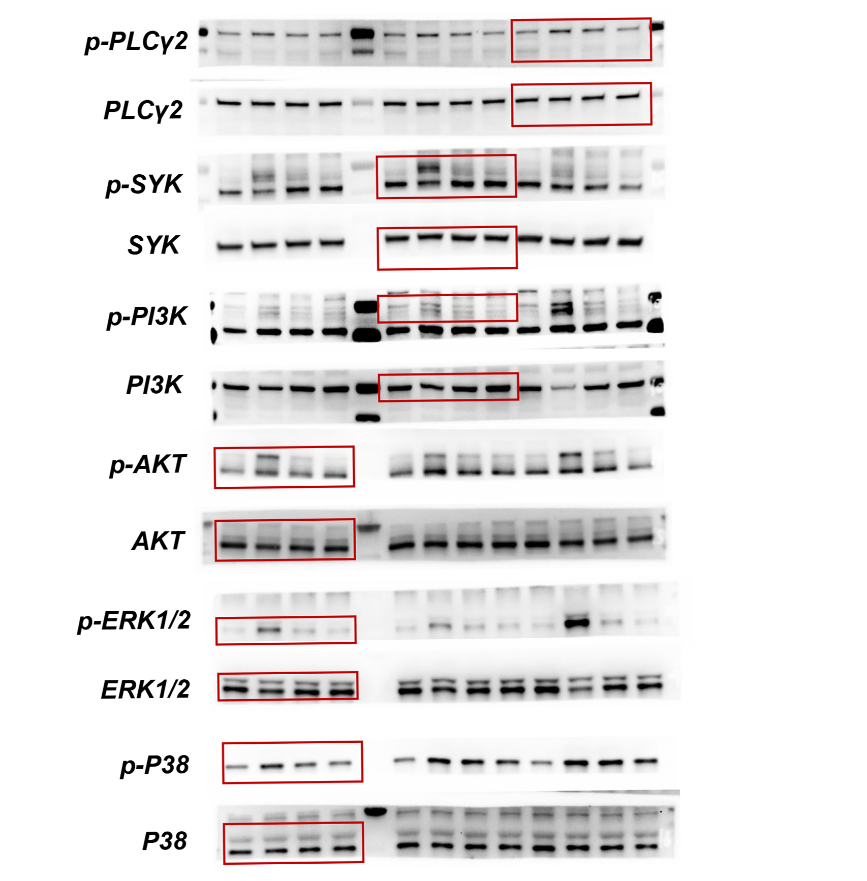

Supplement: Supplementary file 2 — Additional file 1. [file 12964_2023_1276_MOESM1_ESM.docx]
